# Physicians and Druggists in America

**Published:** 1870-10

**Authors:** 


Physicians and Druggists in America.—Dr. A. B. Prescott, of Ann Arbor, Michigan, states that there were 55,000 physicians in this country at the last census, and 11,000 druggists. At present there are about 74,000 physicians.

